# Chorea and Whooping Cough

**Published:** 1880-05

**Authors:** A. R. Smart

**Affiliations:** Hudson, Mich.


### Selections.

---

### CHOREA AND WHOOPING COUGH.

A. R. SMART, M.D., HUDSON, MICH.

I cite the following history of a case of whooping cough, not for the purpose of discussing that disease, but to call attention to a phase of that portean malady, chorea, and to notice its relationship to pathological conditions with which it may be associated, appearing more often, perhaps, as an idiopathic disease. Yet chorea is often found as a result or complicating factor of some other malady, as, for instance, rheumatism with or without pericarditis or endocarditis, pneumonia, paralytic conditions, etc. Its mode of origin and its exact relation to some of these states is obscure and oftentimes ill-defined as in this case, whose history is briefly—a healthy, robust child of 18 months, was found, after the usual prodroma of catarrhal symptoms, to have whooping cough. No exposure to the disease had been known to occur, and nothing unusual was noticed about the case until the convulsive stage had lasted about two weeks, when after an exposure to a cold wind in riding, the child was noticed to be feverish and drowsy, did not take its food, with other symptoms, indicating some graver disease than simple whooping-cough. Examination of the chest revealed diffuse bronchitis, most developed on the right side; the child was also ascertained to be teething, the gums of both the upper and lower iaw being found swolen and tender; poultices were applied to the chest and the treatment modified to relieve the chest symptoms. During the next week following the child continued without apparent material change, a moderate intestinal catarrh developed, causing some diarrhea with mucous and vitiated motions. Suddenly a severe convulsion occurred, lasting about one hour, and as usual, followed by a prolonged drowsiness and stupor, the eyes remaining strabismic a number of hours. This eclamptic seizure was treated by ice to the head, per cartharsis, and the administration of pot. brom. with gelsemium and ergot. After the effect of the convulsion had passed off, the febrile phenomena, chest symptoms and general condition of the child continued for another week with but little change, all but one of the teeth coming through during the time, when convulsions again came on. The onset of the convulsive seizure was not so abrupt as before, but they continued to recur with varying severity, yet without any intermission for about twelve hours, gradually ceasing; they were more severe on the right side, the right eye was more distorted than the left; the attack was followed by complete loss of consciousness and stupor which continued for over two weeks. For some days the eyes were distorted, turning upward and inward, the lids closed and pupils dilated, but movable. After the convulsions ceased the right arm and limb were found to be nearly powerless, the child could move them a little but would not do so if undisturbed. The cutaneous sensibility of the side seemed but little affected while in this condition. A few days after the convulsions symptoms of lobular pneumonia appeared, the coughing fits had continued to recur with increasing frequency and violence, the sputa now were occasionally rusty colored, for two or three days the expectoration was muco-purulent, one day being especially fetid, offensive and profuse. Gradually and slowly the chest symptoms began to lessen, the cough is now less frequent and severe, the stupor is lessening, the eyes are straight but seem wholly insensible to light, the pupils are moderately dilated and respond sluggishly to the action of strong light, but when the child's eyes are wide open, the hand or any bright substance passed quickly before the eyes elicits no response or token of vision, the paresis of the arm and leg is now disappearing, the child can use them moderately well, especially the leg. As the muscular power returns choreic motions are noticed in the arm, followed a little later by choreic movements of the eye lids and eye balls, and of the tongue and lips, slight motions also are noticed in the facial muscles, the hearing seems greatly impaired as well as vision. During this time while these phenomena were transpiring, the child took its food well, even greedily at times, the bowels had become normal, the febrile symptoms are not continuous, but recur from time to time without regularity, the choreic symptoms lasted nearly two weeks, more or less constant. During the severity of the motions he presented a most grotesque appearance. His eyelids were in constant motion, he executed a spitting movement with profuse expectoration of saliva; he makes at times various grimaces; as the movements of the mouth and eyes become more pronounced, those of the arm lessen in severity; at times he seemed unable to swallow, probably from the lack of control of the tongue; the movements all cease while he sleeps, which he does for two or three hours at a time. While awake they will continue for hours in succession. Slowly the cough began to abate, the febrile symptoms ceased, the chest cleared up, the choreic movements subsided somewhat abruptly, the visual power slowly returned and he began to notice things done and said about him, and ultimately the boy made a complete recovery.

With this history let us very briefly discuss the import of the phenomena presented in this case, and inquire what relation may have existed between them. As factors of the pathological problem we have—1st. A convulsive neurosis in the attack of whooping cough, the effect of which is greatly to increase the reflex excitability of the excito-motor ganglia of the cord and brain. Incident to this is the protracted interference with the circulation, the oft-repeated conjestion which must seriously affect the nutrition of the nerve centers. 2d. Dentition, which is well known to be coincident with increased sensitiveness of the nervous system and with intestinal disorder, or at least with unusual disposition to follicular disturbances which increase the reflex excitement of the nerve centres. 3d. The inflammatory chest disease, of which chorea is said to be an occasional complication. It would also be interesting to know the precise locality and nature of the lesion causing the eclamptic attack and subsequently the chorea. Convulsions are not a rare event in whooping cough occurring, according to different observers, in from 1-5 to 1-17 of the cases. They arise for the most part from the repeated shocks conveyed to the excito-motor centres in the base of the brain along the tract of the pneumogastrics. The principle or poision which causes whooping cough, excites by an elective affinity these nerves, yet it gains an entrance to the blood by the pulmonary tract and may act directly upon the respiratory ganglia in the brain. However this may be, gross lesions are seldom found after convulsions of whooping cough, and usually no permanent structural mischief remains. No constant anatomical change in the brain is characteristic of whooping cough, nor is their any peculiar, fixed state of the blood, such as occurs in rheumatic fever or other conditions attended with hyperinosis states of the blood favoring the occurrence of embolism or thrombosis. Each coughing fit involving the laryngeal and entire group of respiratory nerves, must excite and disturb the centers from which these nerves arise. Marshall Hall thought spasm and closure of the larynx essential to a convulsive seizure. Then again their nutrition must suffer from the stasis and venous engorgement incident to the respiratory spasm. The irruption of the teeth and the development of the follicular structure of the intestine occur at a period of infantile life when the brain is in advance in size and development of the rest of the body and is disproportionately excitable. Reflex causes disturb its balance of normal function with wonderful facility. The case in question had a good share of intestinal irritation and of course suffered the reflex brain disorder common to such conditions. Bronchitic inflammation lent its influence to excite the circulation and increase the vascularity of the nerve centres. Every nerve filament involved in the bronchial membrane, the defective æration of the blood, the increase of effete matter in the circulation, all lend their influence in deranging the function of these centres. Thus much for the causation. What was the seat of the lesion causing the convulsions? The nerve distribution involved in a paroxysm of whooping cough is derived from the pneumogastric, through its laryngeal and pharyngeal, bronchial and tracheal branches; the pneumogastric arises from the restiform and pyramidal bodies in the medulla, this tract is directly continuous with the floor of the fourth ventricle and with the motor tract in the pons varolii. Whether the spasmodic action in this tract of nerve supply is peripheral or centric in origin is of no consequence to our inquiry. Repeated excitement and stimulation of the excito-motor centres would disturb their nutrition and function. From the same locality arise also the motor-oculi or 3d nerve supplying the internal and superior recti and the levator palpebræ; the hypoglossal, the motor nerve of the tongue, has an origin near that of the pneumogastric, and was both involved in the cause and later on in the results of the spasms. The lesion, whatever its nature, extended upward so far as to involve the corpora striata, which are believed to be the motor centres for arms and legs. "When the deeper portions of the corpora striata were irritated, the animal alternately put out and drew in its tongue," is the report of some experimenters on animals. The motions of the tongue were much disturbed in this case.

As to the nature of the lesion in the brain, we may infer that the first convulsive seizure was the result of congestive over-distension of the vessels of these centres, causing pressure on the nerve tissue, probably excited by the continued stimulation of the periphery of the nerve trunks having an origin there. The attack soon passed off, leaving no apparent results. The turgid condition of the blood vessels, however, continued until the onset of the second seizure. This was more gradual in development than the first, and was most severe on the right side, clonic spasms continuing on that side for a number of hours. Upon their subsidence, the right arm and leg were nearly powerless, and the visual power wholly gone. We may infer that the vessels involved, strained to their utmost tension, and the capillaries of the parts over-distended, suffered at points a rupture of their walls with small extravasions into the nerve tissue. These lesions of the capillaries occurred mainly on the corpus striatum of the left side, and extended downward into the motor tract of the pons varolii. Thus may be explained the protracted but not severe spasms. After some time had elapsed, and the extravasion had begun to clear up, the perverted and enfeebled nutrition of the structure involved gave rise to the choreic movements, first in the arm and leg, then in the eyelids, tongue and muscles of the face. The grimaces were entirely confined to the eyelids, tongue and muscles of the lower part of the face. This will readily be understood, by remembering that the motor root of the trifacial has its origin in the motor tract of the pons contiguous to that of the motor oculii. If the nature of the brain lesion had been simply prolonged, passive dilatation, the duration of the results would not have been so long, nor would they have been unilateral. Had vernacelar effusion occurred from the protracted and increased vascularity—a reasonable conjecture—the symptoms would have been bilateral, retraction of the head and vomiting might have been expected ; rupture of a larger vessel would have occasioned more complete and lasting paralysis. Thus we are led to conclude that the lesion in the second instance was capillary hemorrhage. If this be a correct view, the case goes far to support the theory advanced, I think, by Dr. Bastian, that chorea is caused by capillary hemorrhage, or embolism, a sufficient interference with the blood supply of the corpora striata to disturb the nutrition, but not to cause paralysis. Hemi-chorea is known to follow in the wake of hemi-plegia, and to result from the impared nutrition of the brain structure. In this case no embolism was probable, as there was no heart inflammation to give rise to it. The most reasonable conjecture seems to be that the second conclusive seizure was caused by, or at least attended with, capillary hemorrhage in the left corpus striatum and into the motor tract of the pons varolii. The optic nerves may have been involved at their origin, which as they wind around the crus cerebri, lie close to the corpora striata, or along their course on one side. The free decussation of their fibres would explain the double effect.

The lesion in the pons may have been only on one side, as the fibre of the motor oculi freely decussate. The fact that the leg was the first to regain its power, also shows the similarity of the lesion to hemorrhage into the corpora striata, causing hemiplegia, in which case the leg is usually first to recover muscular power.

